# Experimental Models and Their Applicability in Inflammation Studies: Rodents, Fish, and Nematodes

**DOI:** 10.3390/ijms26135987

**Published:** 2025-06-22

**Authors:** Ana Emilia Nascimento Lemos, Jaluza Luana Carvalho de Queiroz, Bruna Leal Lima Maciel, Ana Heloneida de Araújo Morais

**Affiliations:** 1 Biochemistry and Molecular Biology Postgraduate Program, Biosciences Center, Federal University of Rio Grande do Norte, Natal 59078-970, RN, Brazil; 2 Department of Nutrition, Health Sciences Center, Federal University of Rio Grande do Norte, Natal 59078-970, RN, Brazil; 3 Nutrition Postgraduate Program, Health Sciences Center, Federal University of Rio Grande do Norte, Natal 59078-970, RN, Brazil

**Keywords:** immune system, inflammatory mediators, animals, laboratory

## Abstract

Experimental models have been widely used to study the mechanisms of inflammation due to their genetic and physiological relevance to humans. These models include rodents (rats and mice), zebrafish, and nematodes (*C. elegans*). Considering the similarities and divergences between experimental models and the human organism, this narrative review aimed to compare and discuss their applicability in inflammation studies. Rodents, in particular, share significant similarities with humans across approximately 85% of their genome, making them ideal for investigating complex diseases and inflammatory responses. Zebrafish also stand out for showing high conservation of the immune system compared to humans, being useful for studies of adaptive and innate inflammation. Despite not having adaptive immunity, *Caenorhabditis elegans* is a robust model for understanding innate immune responses, especially in studies involving host–pathogen interactions. These organisms allow us to efficiently investigate the acute and chronic phases of inflammation, offering an accessible platform to study complex biological processes that are unfeasible in humans due to ethical and financial constraints. Thus, the use of these models has been essential for inflammation research. However, the use of each one will depend on the research question and hypothesis raised.

## 1. Introduction

Inflammation is a component of the immune response to injury, infection, and other harmful conditions that compromise cellular or tissue integrity. This standard response is common to several types of tissues and is mediated by several substances produced by damaged cells and by cells of the immune system. It is characterized by two phases: acute and chronic. Acute inflammation has been considered a key component of innate immunity and the primary line of host defense in response to injury or the invasion of pathogens. However, when the inducing agent persists over time and is not eliminated, the inflammatory response can become chronic, with devastating consequences [1,2,3].

Thus, inflammation is an essential response generated by the innate or adaptive immune system of all living organisms, from the simplest to the most complex, and is considered a protective reaction to various external stimuli, such as invading pathogens, cell or tissue damage, and irritants [4,5]. However, innate immunity presents nonspecific protection mechanisms that do not differentiate between external stimuli. It is the evolutionary part of the immune system with the ability to guarantee immediate protection against external stimuli and is present in most invertebrate animals [6]. Adaptive defense emerged only during the initial evolution of vertebrates and involves defense strategies that adapt to each invader. Furthermore, adaptive response has only been attributed to vertebrate animals [7]. However, even in vertebrates, adaptive immunity is strongly dependent on the activity of innate immune cells, and the evolution of these immunities forms a diverse and efficient immune system present in mammals [6].

The innate immune system in humans consists of various cells, including neutrophils, monocytes, macrophages, and immature dendritic cells, known as phagocytes. These cells have a dual function in immunity: they eliminate non-self molecules and pathogens while also processing self-antigens derived from engulfed apoptotic cells [8,9].

Furthermore, in adaptive defense, cellular or tissue damage in vertebrates can promote the recruitment of inflammatory cells and cytokines, inducing inflammation [7]. This inflammatory process begins activating special receptors, such as damage-associated molecular pattern detection receptors or pattern recognition receptors (PRRs), due to antigen entry or tissue damage into the body [10].

Among the factors recruited in inflammation are lymphotoxins, interleukins, chemokines, and interferons. When released, these factors can act on the cells that secrete them as an autocrine signal or on different cells as a paracrine or endocrine signal. However, when binding to specific receptors, these factors activate cascades of signaling pathways studied in several diseases, including inflammatory disorders [11].

As already mentioned, among the inflammatory cytokines studied, there are the *tumor necrosis factor-alpha* (TNFα) and the *interleukin-beta* (IL1β), which is also an important initiator of inflammation [12], in addition to other cytokines such as *interleukin-6* (IL-6), and also adhesion molecules, proteolytic proteins, histamines, prostaglandins, leukotrienes, neuropeptides, and neurotransmitters that play important roles in the inflammatory process [13]. In this inflammatory process, common to the adaptive immune response, the regulation of the genes that encode all these mediators, already mentioned, is only possible with the activation of transcription factors [14]. It is important to highlight the role of the transcription factor of *nuclear family factor kappa B* (NF-κB) in the modulation of gene expression and the release of inflammatory cytokines due to their high conservation in vertebrates, from mammals to fish [15]. Given the importance and the most diverse mechanisms involved in the inflammatory response, experimental models that have genetic similarities to humans have been used for years to study this response’s evolution in the acute and chronic phases of inflammation. These models, which have answered the most varied biological and medical questions unfeasible to test in clinical trials, due to the cost and complexity of the system, and ethical issues, have been used as a strategy to better understand the mechanisms of inflammation in the face of the most various stimuli [16,17].

Therefore, among the animals used in research related to inflammation are rodents (rats and mice). These animals have been considered applicable due to their physiological and genetic similarities to humans, making them a good model for studying complex diseases, in addition to being relatively easy to handle and transport [18]. Among fish, is the zebrafish, which shows extreme similarity to humans in terms of the composition, function, and molecular mechanisms of the immune system [19]. Among the nematodes used as experimental models, the highlight is *Caenorhabditis elegans* (*C. elegans*) as a robust model used to study pathogenic immune responses to bacteria and immune defense mechanisms, as it has a conserved immune system [20].

Thus, this review presents a compilation of information on the experimental models used to investigate various scenarios within the inflammatory process. These models are an essential part of research as it encompasses medical and biological issues that cannot be studied in humans. These experimental models have genetic similarities to humans, which may facilitate the pursuit of promising results related to inflammation. Therefore, this narrative review aimed to compare three experimental models and evaluate their applicability in inflammation-related studies.

## 2. Experimental Models for Evaluating the Inflammatory Response

### 2.1. Rodents

Among various animal models, rodents, especially mice and rats, are frequently used in experiments due to their biological characteristics [21]. Because rats and mice share strong similarities with human physiology, their use in preclinical research is an invaluable tool for enhancing the understanding of human diseases and supporting the development of new therapeutic strategies [22].

Due to their similarity to humans, rats have become preferred in genetics and genomics research. They share approximately 85% of their genome with humans, making them an ideal model for studying genetic factors related to human health and disease. For this reason, scientists can investigate the molecular causes of various diseases, identify hereditary risk factors, and develop therapies more effectively [23].

Additionally, rats reach sexual maturity quickly (less than 8 weeks after birth) and have a relatively short reproductive cycle and gestation period, approximately 3 weeks [21]. Moreover, they have a relatively short lifespan, as the entire life cycle of these animals can be studied within two to three years [24,25].

Meanwhile, mice are considered the most studied mammals in human diseases and biological research due to their physiological, anatomical, and genetic similarities to humans, along with their advantages, such as a short lifespan and gestation period (18.5 to 21 days), high fertility (2 to 12 or more offspring), and sexual maturity at five to eight weeks, making them favorable for many types of studies [26].

Studies show that the genetics of mice are essential for assessing the causal relationship between genetic variables and the onset of diseases, given that this experimental model offers unique opportunities to dissect biological mechanisms and systems in vivo, providing a deeper understanding of disease pathophysiology and development. This ranges from basic mechanistic studies to preclinical investigations, the identification of therapeutic targets, and the development of new therapeutic interventions [27].

The mouse is an experimental model with several analogies to the human organism, particularly regarding systems involved in the inflammatory process. Among these is the regulation of neutrophil chemotaxis, which involves the same subtypes of purinergic receptors (P2Y2, A3 e A2a) for autocrine signaling. The authors also mention the nuclear activation of the NF-κB in pulmonary and peripheral neutrophils after stimulation with endotoxin, as well as the similarities in the detection of molecular patterns associated with pathogens via receptors *Toll-like* (TLRs). Moreover, the activation of the NF-κB induced by IL-1 promotes the transcription of the gene *mlck*, associated with increased intestinal permeability, reinforcing the similarities between the inflammatory responses of this model and the human organism [28].

Additionally, rodents can be inbred, allowing the production of genetically identical strains that enable the study of transgenerational effects and effects on single genes through the development of transgenic or knockout animals, facilitating the study of a wide range of diseases [24,25]. However, it is important to note that although research with animals serves as a guide for subsequent clinical experiments, a limitation is that, since inbred animals are genetically identical, they lack heterogeneity in the human population [28].

It is important to emphasize that the most relevant differences between inbred and outbred animals are, in fact, genetic. Inbred animals have a highly homogeneous genetic background characterized by fixed alleles that result from successive generations of crosses between closely related individuals. This genetic uniformity reduces phenotypic variability, allowing for greater experimental control, but it also limits adaptive responses to environmental disturbances. In contrast, outbred animals maintain greater genetic variability, with multiple allelic variants distributed across several biological pathways, which confers greater phenotypic robustness in the face of environmental or experimental changes. This genetic diversity present in outbred animals can act as a compensation mechanism, stabilizing phenotypic responses and increasing the external validity of experimental findings. Therefore, although physical differences, such as body weight, are often observed, it is the genetic heterogeneity of outbred animals that constitutes the primary functional distinction between the two models, especially in the context of biomedical research and experimental reproducibility [29].

Table 1 presents the main similarities and differences between rats and mice, highlighting their contributions and limitations in the study of complex diseases and the investigation of biological mechanisms.

### 2.2. Zebrafish (Danio rerio)

The zebrafish (*Danio rerio*) has been used in various research fields, including genetics, toxicity, and biology. It has become a relevant vertebrate animal model due to its homology with over 70% of human genes. Furthermore, most of the pathways, cell types, and tissues involved in human diseases are conserved in the zebrafish [30].

Among other advantages of the zebrafish, its size, ex utero development, optical transparency, high fertility, and ease of genetic manipulation are worth highlighting, as they favor translational responses for vertebrate organisms such as humans. Additionally, it has proven to be an experimental model with solid potential for use in testing to discover new therapeutic agents [31].

Regarding the study of inflammation, the zebrafish has some adapted protocols that have been successfully used to assess the response during the progression of the inflammatory reaction [32]. The inflammatory response in zebrafish effectively replicates the inflammatory process in mammals. Based on this, some studies provide evidence that immune signaling pathways and gene expression are well conserved throughout evolution [33].

**Figure 1:**
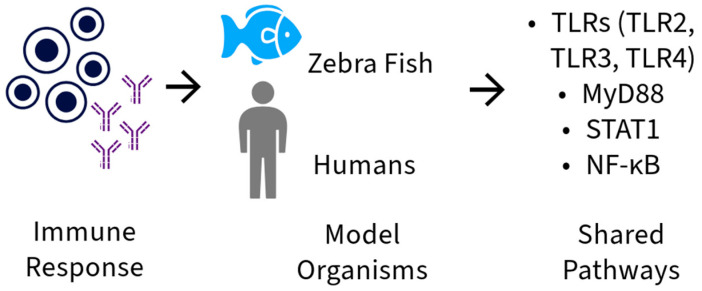
Similarities in immune response pathways between zebrafish and humans, highlighting receptor activation Toll-like: Toll-like receptor 2 (TLR2), Toll-like receptor 3 (TLR3), Toll-like receptor 4 (TLR4), and their myeloid differentiation factor signaling pathways 88 (MyD88), transcription activator signal transducers 1 (STAT 1) and Nuclear Factor Kappa B (NF-κB).

**Figure 2:**
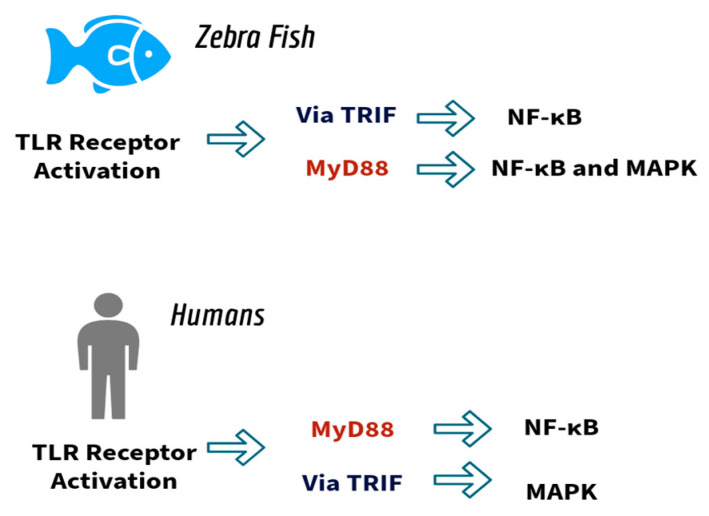
Differences in inflammatory signaling pathways between zebrafish and humans, signaling pathway of the interferon-β (TRIF), Nuclear Factor Kappa B (NF-κB), myeloid differentiation factor 88 (MyD88), and mitogen-activated protein kinase (MAPK).

TNF-α plays essential roles in maintaining homeostasis and in the pathogenesis of inflammatory diseases, making it crucial for host defense [34]. IL1β is an essential initiator of inflammation in response to injuries and infections [11,35,36]. The genes of these cytokines, *tnf-α* and *il1β*, are well conserved between zebrafish and mammals. Additionally, most components of the signaling pathways involved in the inflammatory process are also present in these fish, including *myeloid differentiation factor 88* (Myd88), caspase 1, and NF-κB, as well as two homologous copies of the gene TNF-α, which are *tnfα1* and *tnfα2* [12]. In terms of immunity, the zebrafish has both innate and adaptive immune systems [37], making humans and zebrafish extremely similar in terms of composition, function, and molecular mechanisms of the immune system [19]. In zebrafish, the genes related to innate immunity are primarily those involved in the signaling pathways of *Toll-like receptors* (TLRs): TLR2, TLR3, TLR4, activated through signaling MyD88 and *transcription activator signal transducers 1* (STAT 1) and NF-κB (Figure 1 and Figure 2) [37].

When the family’s expression is activated, TLRs present in the immune cells of zebrafish trigger the signaling pathways of interferon-β (TRIFI). In this specific case, TLR3 can initiate pathways dependent on TRIF, and the other members of the family of TLRs have the ability to mediate the expression of MyD88 [38].

As mentioned, the *mitogen-activated protein kinase* (MAPK) and the NF-κB are two classic inflammatory signal transduction pathways downstream of the pathways dependent on MyD88 and TRIF. Thus, TRIF can mediate the signaling pathway of NF-κB, as well as that of NF-κB 2 and p65, which are considered important members of the NF-κB family. However, the activation of TLRs/MyD88 not only induces the signaling pathway of NF-κB but also activates the signaling pathway of MAPK, which plays an important role in the regulation of cell growth, migration, senescence, and autophagy [19]. In turn, the imbalance of the signaling pathway of the TLRs can stimulate the activation of the innate immune system in zebrafish, leading to abnormal immune system function [39,40,41,42].

Therefore, TLRs are a crucial pathway for the induction and progression of several diseases, considered one of the receptors of *pathogen-associated molecular patterns* (PAMPs), used to detect pathogens in the immune system, and to assess the host’s immune defense against pathogens [43].

The activation of immune cells is a common initial inflammatory response in both mammals and zebrafish following an injury. However, while in zebrafish, this response promotes tissue regeneration by stimulating stem cell activity, in mammals, it tends to limit regenerative capacity, hindering the repair process. Therefore, analyzing the role of each type of innate immune cell and uncovering their molecular signaling during the regeneration response will provide important insights for human therapies [44].

In this way, the zebrafish presents unquestionable advantages compared to other vertebrate models related to the aforementioned biological characteristics, development, physical aspects, and genetic manipulation, which facilitates the interpretation of findings [45]. Moreover, zebrafish have regulatory mechanisms for the genome and proteins that are highly homologous to humans, and they also possess intact innate and adaptive immune systems [37].

### 2.3. Caenorhabditis elegans

*C. elegans* is considered a highly suitable and versatile in vivo model, exhibiting several characteristics that facilitate experimentation, as well as having fully sequenced genes that are largely homologous to human genes, sharing 60–80% similarity at the genomic level [46,47]. Additionally, they have a transparent body and a short lifespan (about three weeks), which, combined with their small size, allows almost all biological processes to be observed and measured. This makes them an ideal system for live and real-time imaging, with regulatory pathways that are highly conserved between *C. elegans* and mammals, including humans [48].

In fact, due to their transparency, the use of *C. elegans* mutants with reporter genes, or when modified with fluorescence, helps in the evaluation of gene expression of various genes or in confirming alterations in signaling pathways [49]. They also present themselves as a model with low maintenance and propagation costs. They can be genetically manipulated both through *Clustered Regularly Interspaced Short Palindromic Repeats* (CRISPR) *associated protein 9* (Cas-9) as well as through *RNA interference* (RNAi) [46]. Moreover, the use of *C. elegans* for experiments does not require approval from the Institutional Animal Care and Use Committee, as *C. elegans* is considered a compatible in vivo model for research, respecting the principles of 3R (*Reduction, Replacement, Refinement*), especially *Refinement*, which aims to refine experimental protocols to minimize animal pain or stress [50,51].

Thus, this model has entered the scientific scene and continues to attract the attention of researchers for helping to unravel the mysteries of vital processes, the pathogenesis and pathology of diseases, the metabolism and pharmacology of drugs, the toxicology of environmental factors, and new therapeutic agents, among other applications [52,53,54]. Therefore, it is considered a reliable model for evaluating a wide range of diseases [55].

As a model for studying inflammation and immunity, *C. elegans* is more similar to an antibacterial model, developed by exposing it to pathogenic bacteria, in which immune regulation mechanisms can only be reflected at the level of innate immunity, as it lacks adaptive immunity [52,56,57]. By living with various pathogens, such as bacteria, fungi, and viruses in the soil, *C. elegans* defend themselves through the cuticle and epidermis, the uterus, and the rectum, or they may colonize the intestine. Since they lack specialized immune cells, these tissues play a role in defense [52].

Considering the study of inflammation, *C. elegans* can be used as an experimental model, as these nematodes provide initial selection and biological theoretical basis for inflammation-induced diseases. On the other hand, there are limitations to using this animal model, as *C. elegans* does not have many organs such as the liver, heart, or stomach. Also, multiple biochemical indicators are not entirely independent, besides having only an innate immune response, as previously mentioned [52], and as a disadvantage, there is the absence of an adaptive immune response [58].

Among the immune signaling pathways in *C. elegans*, three main pathways stand out: the signaling pathway of *growth transformation factor beta* (TGF)-β, the signaling pathway *mitogen-activated protein kinase signaling pathway* p38 (p38 MAPK), and the signaling pathway of *insulin-like growth factor type 1* (IGF-1). These play different roles in resistance to external adverse stimuli, respectively [59].

TGF-β in *C. elegans* has been regarded as a simplified version, which may facilitate a better understanding of the multifunctional bases of this signaling pathway. In addition to being one of the best-known TGF-β pathways for controlling body size and male tail development, it also has the ability to regulate other phenotypes later identified, such as immune response, longevity, reproductive period, and matricide [60].

The signaling pathway of p38 MAPK is highly conserved in mammals and is involved in responses to various physiological stimuli and environmental stresses, in addition to playing an important role in the intrinsic immunity of these nematodes [21]. The insulin-like signaling pathway is an evolutionarily conserved pathway with significant functions in phosphorylation, well-known for regulating metabolism and growth throughout life. It regulates immune responses in *C. elegans* [61].

Thus, in *C. elegans*, metabolism influences immunity, and this occurs through the regulation of nutrient levels via the pathways of p38 MAPK and *activating transcription factor* (ATF-7)/CREB. This is also influenced by the *transcription factor forkhead* (DAF-16/FOXO), which reduces food intake, thus demonstrating a molecular link between digestion, growth, and innate intestinal immunity in these nematodes [62].

IGF-1 is a growth hormone in humans and is found in *Caenorhabditis elegans*. In *C. elegans*, it is primarily regulated by the *DAF-2* gene, which functions similarly to the human insulin receptor, and the *DAF-16* gene, which is related to FOXO transcription factors found in humans. These genes are fundamental for the molecular processes of IGF-1. The insulin signaling pathway, involving the interaction between *DAF-2* and *DAF-16,* is one of the main mechanisms regulating glucose transport and insulin response. Therefore, this pathway is extensively studied in research on insulin resistance and type 2 diabetes [63].

According to Mchugh [64], aging in *C. elegans* decreased the activity of the PMK-1/p38-ATF-7/CREB pathway, which plays a significant role in the immune response. Because of this reduction, insulin signaling became more prominent, indicating a shift in immune and metabolic processes as the organism ages. Thus, with aging, levels of insulin-like peptides, such as INS-7, increase, leading to the activation of the DAF-2/ILR receptor and the negative regulation of DAF-16/FOXO activity.

Interestingly, DAF-16/FOXO exerts negative control over INS-7, and this increase in INS-7 expression is enhanced by the action of bZIP transcription factor ZIP-10 [65]. In addition to regulation mediated by insulin-like peptides, a growing body of evidence suggests that the neurotransmitter GABA also plays a role in modulating the DAF-16/FOXO pathway [66] and the PMK-1/p38 pathway. The latter appears to play a central role in integrating immunity and longevity in *C. elegans*, linking the immune response to aging. These regulatory mechanisms, involving feeding, immune defense, and aging, reflect the variable conditions of this organism’s natural environment and allow it to adjust the balance between survival and longevity. This adaptive process may be conserved in other species, including humans, in the regulation of immunity and aging [67]. However, *C. elegans* models offer an opportunity to study immunity or inflammatory processes.

## 3. Anti-Inflammatory, Inflammation, and Experimental Models

Several bioactive components in foods and botanical products are anti-inflammatory and represent appealing means to treat and/or prevent inflammation [68]. Thus, understanding how these anti-inflammatory components can modulate intracellular signaling is essential for identifying specific molecular targets and validating their use as new therapeutic agents [69].

The mouse is one of the most reliable models for studying immune-mediated inflammation in anti-inflammatory drug research, as it more effectively simulates the pathological characteristics of inflammatory diseases. Thus, animal experiments are divided into acute and chronic inflammatory models, and numerous studies have used rats and mice to investigate inflammation in traditional Chinese medicine [70].

Rats are used in experimental models of inflammation, as they allow for the evaluation of chronic inflammation, which is associated with increased levels of inflammatory cytokines (such as TNF-α, MCP-1, NF-κB p65, IFN-γ, IL-2, IL-17, IL-1β, IL-6) and elevated numbers of neutrophils and macrophages [10].

Zebrafish are commonly used to evaluate foods’ anti-inflammatory effects, components, and medications because their immune system contains nearly all the same cell types as the human immune system [38,71].

*C. elegans* can be used to evaluate a wide range of anti-inflammatory components; however, other models (such as zebrafish or rodents) are often preferred for studies specifically involving humoral or cellular immunity. In contrast, *C. elegans* is particularly suited for understanding host–pathogen interactions [52] (Table 2).

In the areas of genomics and genetics, rodents are preferred over zebrafish (*Danio rerio*), as they have essential physiological and genetic similarities with humans and have, therefore, become a good model for studying complex characteristics and diseases, as well as systemic inflammatory diseases and the preclinical validation of therapies. This is due to the high genetic homology that rodents have with humans, as well as the presence of a complete immune system (innate and adaptive) [21]. Furthermore, rodents also have a functional TLR4 receptor essential for inflammatory responses mediated by lipopolysaccharides (LPSs). They are considered indispensable in studies involving acute and chronic inflammation, including models of sepsis, colitis, rheumatoid arthritis, and experimental autoimmune encephalomyelitis [72].

Despite the limitations presented by *C. elegans*, it has been used as an experimental model to study metabolic disorders associated with inflammation, such as obesity and insulin resistance. *C. elegans* presents the capacity for lipid storage and conserved metabolic pathways, which allows the investigation of the effects of omega-3 polyunsaturated fatty acids in the modulation of inflammation and lipid metabolism [73]. Furthermore, this model is also used to understand immunosenescence due to its simple immune system and rapid aging process, presenting the activation of the p38 MAPK pathway, together with transcription factors such as SKN-1/NRF and DAF-16/FOXO, which plays a significant role in the regulation of the immune response during aging in *C. elegans* [74].

## 4. Conclusions and Perspectives

This review focused on the diversity of experimental models available to investigate inflammatory processes and their feasibility for testing anti-inflammatory molecules in vivo. Exploring and developing various inflammation-related models is crucial to bridging the gap between traditional animal studies and human biology. Models that resemble the human microenvironment are essential to understanding the complexities of the inflammatory process and immune response. As presented in this review, these experimental models allow the investigation of various situations occurring in the inflammatory process, becoming a crucial part of biomedical research as they address fundamental biological and medical issues that would be impossible to study in humans due to cost, complexity, and ethical concerns.

On the other hand, these models provide an unparalleled opportunity to understand molecular and cellular levels. They offer crucial insights into how innate and adaptive immune mechanisms and the inflammatory process are linked to the organism’s physiology.

Based on the similarities observed between mice and humans regarding regulating the inflammatory response, rodents offer significant advantages when used as an animal model in experimental studies. Among these advantages is that mice share crucial mechanisms, such as regulating neutrophil chemotaxis, the activation of NF-κB, after stimulation by endotoxins, and pathogen detection patterns mediated by TLRs. Rats share similar mechanisms of chronic inflammatory response, such as the increase in key inflammatory cytokines (TNF-α, MCP-1, IL-1β, IL-6, IFN-γ, NF-κB p65, among others) and immune system cells such as lymphocytes and macrophages.

These similarities, combined with the ease of genetic manipulation and the short reproductive cycle, make mice a valuable tool for experimental studies. They allow the extrapolation of results to human conditions and contribute to the advancement of understanding inflammatory mechanisms and the development of therapies. The zebrafish has emerged as an animal model with a short life cycle and easy maintenance, making it an attractive alternative for studies on inflammatory diseases, thanks to its optical transparency. Furthermore, the high similarity of zebrafish inflammatory genes with those of mammals is essential for investigating the mechanisms of inflammatory diseases. In this way, some studies have shown that inflammatory signaling in zebrafish is similar to that in mammals, validating it as an ideal model for analyzing inflammation-related diseases [75].

Some authors provide evidence that the same signaling pathways between zebrafish and humans often play instructive and beneficial roles in drug discovery. Therefore, zebrafish have been used to study various human diseases, contributing to many important discoveries with translational significance [30,76]. The zebrafish offers a powerful set of tools to answer various questions, with a unique and reliable system for inflammation-related studies.

As for *C. elegans*, despite the many advantages presented, it has its limitations regarding the absence of some organs. As a nematode, it is biologically distant from mammals, which reduces its ability to predict various biological events in humans. Additionally, many molecular pathways in mammals do not exist in nematodes, which may limit some studies. Therefore, *C. elegans* should be seen as a valuable model to elucidate mechanisms of action and as a rapid screening system for early-stage research, such as in establishing the function of a gene or obtaining preliminary information that facilitates more detailed preclinical developments in other, more complex animal models [77].

However, to evaluate humoral or cellular immunity, zebrafish or rodents may be more appropriate, while the model of *C. elegans* may be more suitable for understanding the host–pathogen correlation [78]. Models such as *C. elegans* and zebrafish can be adopted for primary or rapid screening, while rodent models are more suitable for in-depth investigations and confirmation of inflammatory processes, thereby supporting the clinical application of findings in the treatment of inflammation [54].

Therefore, experimental models are considered viable and are well-established choices for preclinical studies [78]. Using animals is still considered a vital tool for obtaining essential information and understanding the pathogenesis of various diseases. In this context, animals that allow disease development quickly, are easy to handle, and share genetic similarities with humans should be prioritized. Additionally, those that exhibit similarity with human immunity, facilitating research, explain why several studies have been conducted using rats, zebrafish, and *C. elegans*, and are considered well-established animal models.

As science progresses, there is a notable increase in studies using animal models for different purposes, such as disease induction and/or discovery of potential treatments. However, although studies with animals are the essential foundation for science, playing a key role in human application, ethics are a crucial factor, seeking to minimize pain and suffering, using an ideal number of animals, thus ensuring a reduction in the suffering of the animals used [8].

## Figures and Tables

**Table 1:** Comparison between experimental rodent models: rats and mice.

Criteria	Rats	Mice
Similarities	Rodents share approximately 85% of their genome with humans and can be bred into genetically identical strains, enabling reproducible studies of human diseases	
Differences	Inbred rodents are genetically identical, presenting a gap from the heterogeneity of the human population Inbred rodents are markedly smaller than outbred rodents	
Use in research	Preferred in genetics and genomics studies due to their high genetic similarity to humans	Used in gene–environment interaction studies and genetic manipulation
Reproduction and life cycle	Short gestation (21–23 days) and sexual maturity before 8 weeks	Short gestation (18.5–21 days) and sexual maturity from 5 to 8 weeks
Immune system	There is conservation of innate and adaptive immune mechanisms, including similarities in epithelial barriers and in the presence of T cells, B cells, and natural killer (NK) cells	Similarities in the activation of the NF-κB and regulation of neutrophil chemotaxis
Limitations	Genetic and age similarities between rats or mice and humans; consanguineous genetic profile limits genetic variability and consequently clinical application	

**Table 2:** Comparison of experimental models: benefits and limitations of rodents, zebrafish, and *C. elegans* in inflammation studies.

Experimental Model	Advantages	Disadvantages
Rodents (rats and mice)	Greater ease in evaluating the pathological characteristics of inflammatory diseases	Inbred rodents have an identical genetic profile, which does not reflect the genetic heterogeneity observed in human populations
	Recommended for evaluating humoral or cellular immunity	Although many studies use young animals, which may limit direct applicability to elderly human populations, there are also experimental models using aged rodents that help address age-related immune changes, such as immunosenescence
	Indicated for further investigations and for confirming the inflammatory process (facilitates clinical application in studies on inflammation)	Because they are created in controlled laboratory environments, they may exhibit physiological responses different from those observed in humans exposed to diverse environmental conditions
Zebrafish	Used to evaluate the anti-inflammatory effect of nutrients, bioactive compounds, and medications	Different inflammatory responses, such as facilitating tissue regeneration
	High similarity of inflammatory genes compared to the human organism	Differences in stem cell activity, which may limit the applicability of results in human therapies
	Easy maintenance and high reproductive capacity	Imbalance in receptor signaling pathways *Toll-like* (TLRs) in zebrafish, which can lead to abnormal functioning of the immune system, creating challenges in interpreting data for human application
	Indicated for evaluating humoral or cellular immunity	Although many immune pathways are conserved, evolutionary differences may create limitations when attempting to fully replicate human inflammatory responses
	Indicated for primary or rapid screening studies	Difficulty in modeling complex human inflammatory diseases, such as chronic or multifactorial diseases
Nematodes (*C. elegans*)	A useful model to elucidate mechanisms of action and as a rapid screening system for early-stage research	Morphological differences, limiting the extrapolation of results to humans
	Indicated for studying the understanding of the host–pathogen correlation	Many molecular pathways present in mammals do not exist in nematodes, which may limit certain studies
	Does not require an ethics committee	Limited innate immunity and absence of adaptive immunity

## Abbreviations

The following abbreviations are used in this manuscript:

PRR	receptors or pattern recognition receptors
TNFα	*tumor necrosis factor-alpha*
IL1β	*interleukin**-beta*
IL-6	*interleukin**-6*
NF-κB	*nuclear family factor kappa B*
Myd88	*myeloid differentiation factor 88*
TLRs	*Toll-like receptors*
STAT 1	*transcription activator signal transducers 1*
PAMPs	*pathogen-associated molecular patterns*
CRISPR	*Clustered Regularly Interspaced Short Palindromic Repeats*
Cas-9	*protein 9*
(TGF)-β	*growth transformation factor beta*
p38 MAPK	*mitogen-activated protein kinase signaling pathway* p38
IGF-1	*insulin-like growth factor type 1*
(ATF-7)/CREB	*activating transcription factor*
DAF-16/FOXO	*transcription factor forkhead*
